# Massive data clustering by multi-scale psychological observations

**DOI:** 10.1093/nsr/nwab183

**Published:** 2021-10-08

**Authors:** Shusen Yang, Liwen Zhang, Chen Xu, Hanqiao Yu, Jianqing Fan, Zongben Xu

**Affiliations:** National Engineering Laboratory of Big Data Analytics, Xi’an Jiaotong University, Xi’an 710049, China; Industrial Artificial Intelligent Center, Pazhou Laboratory, Guangzhou 510335, China; National Engineering Laboratory of Big Data Analytics, Xi’an Jiaotong University, Xi’an 710049, China; Department of Mathematics and Statistics, University of Ottawa, Ottawa, ON K1N 6N5, Canada; National Engineering Laboratory of Big Data Analytics, Xi’an Jiaotong University, Xi’an 710049, China; Center for Statistics and Machine Learning, Princeton University, Princeton, NJ 08544, USA; National Engineering Laboratory of Big Data Analytics, Xi’an Jiaotong University, Xi’an 710049, China

**Keywords:** massive data, clustering, psychological observation, Weber–Fechner law, cognitive interpretability, computational scalability

## Abstract

Clustering is the discovery of latent group structure in data and is a fundamental problem in artificial intelligence, and a vital procedure in data-driven scientific research over all disciplines. Yet, existing methods have various limitations, especially weak cognitive interpretability and poor computational scalability, when it comes to clustering massive datasets that are increasingly available in all domains. Here, by simulating the multi-scale cognitive observation process of humans, we design a scalable algorithm to detect clusters hierarchically hidden in massive datasets. The observation scale changes, following the Weber–Fechner law to capture the gradually emerging meaningful grouping structure. We validated our approach in real datasets with up to a billion records and 2000 dimensions, including taxi trajectories, single-cell gene expressions, face images, computer logs and audios. Our approach outperformed popular methods in usability, efficiency, effectiveness and robustness across different domains.

## Teaser

Inspired by psychological principles, the authors designed a big data clustering algorithm with publicly available codes, which can scale to billion-level datasets and is highly effective across various domains.

## INTRODUCTION

Clustering is the discovery of unknown grouping structure in data in an unsupervised way and is a long-standing fundamental problem in data science and artificial intelligence. During the last century, small-scale clustering analyses (typically <1000 records) have been widely used in science, medicine, engineering, economics and humanities [1–7]. Nowadays, datasets with a million or more records are increasingly available in all areas of human endeavors, providing remarkable scientific insights.

Massive datasets are prone to exhibit significant hierarchical structures, reflecting the hierarchical nature of our world. Identifying hierarchical meaningful clusters is essential for massive data clustering, such as building cell atlases with single-cell RNA sequencing (scRNA-seq) data [8,9]. However, most available approaches [10] are computationally unscalable, while the few scalable ones (e.g. *k*-means [11]) suffer from various limitations, including flat clustering assignments, requiring a given cluster number, sensitivity in parameter tuning and ineffectiveness on high-dimensional data. These limitations make clustering a bottleneck of current large-scale data-driven scientific research [9,10].

We aim to systematically design a universal algorithm to simultaneously achieve the following four objectives that are highly desired by massive data clustering: (i) interpretability—the clustering process of the algorithm should be interpretable to better understand and validate clustering results; (ii) high scalability—the algorithm should easily scale to massive datasets; (iii) universality—the algorithm should be effective for various tasks without any prior assumption; and (iv) user friendliness—the algorithm should be very easy to use in practice.

**Figure 1.**
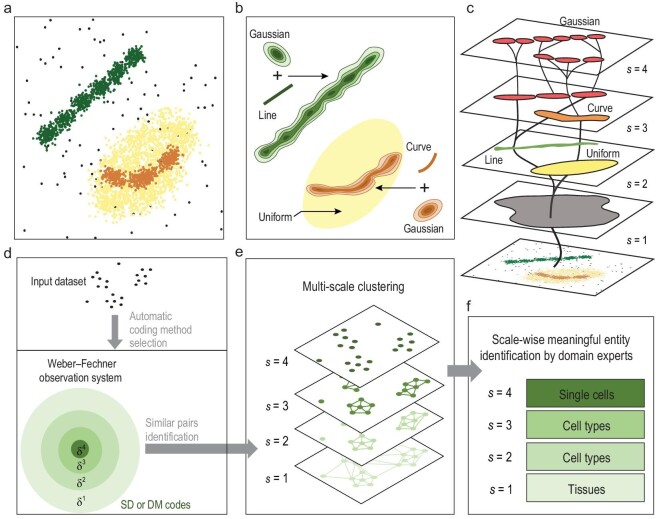
Illustrations of multi-scale clustering of WFC. (a) 3000 synthetic two-dimensional data points. (b) Mixed distributions (grouping structures) hidden in the 3000 data samples. (c) WFC captures emerging clusters as observation scale increases (${\rm{\lambda \ }} = {\rm{\ }}1$). At scale 2, uniform (mixed with curve and Gaussian) and line (mixed with Gaussian) distributions were separated. Curve (mixed with Gaussian) distributions were detected at scale 3. Finally, at scale 4, all clusters representing Gaussian distributions were detected. (d–f) The computation process of WFC, where${\rm{\ }}{{\rm{\delta }}^{\rm{s}}} = {\rm{\ }}1/{\rm{sim}}_{{\rm{min}}}^s$ represents the corresponding distance threshold at each scale. (d) SD or DM coding enables fast computation of similarities for all pairs of data points. (e) Similarities at different thresholds (scales) form multiple connected graphs, each connected component representing a scale-wise cluster. (f) Interpretations of clusters at hierarchical scales according to domain knowledge.

To this end, we design an approach called ‘Weber–Fechner Clustering’ (WFC), by simulating the multi-scale observation process of humans with the Weber–Fechner law [12,13] in psychology. Humans perceive objects in the world and regard them as meaningful entities (e.g. cells and organs) only over a certain range of scales, and different grouping structures emerge as the observation scale changes. Similarly, WFC observes a dataset (digital representations of real objects) and captures the emerging clusters (potentially meaningful entities) gradually, from the grossest scale $s\ = \ 1$ to the finest one ${s_{{\rm{end}}}}$. Figure 1a–c provides an example showing how WFC identifies hierarchically overlapping clusters (mixed distributions) over scales.

The problem here is how to define and update scales to ensure a reasonable finite total number of scales and no information loss between scales. Our previous work [14] adopts scale-space theory to precisely model the scale changing process, but this is computationally prohibitive for massive data clustering. Alternatively, WFC updates scales ${\rm{\Delta }}s\ = \ {\rm{\lambda }}s$ by using the concept of just noticeable difference (JND) in the Weber–Fechner law, i.e. the ratio ${\rm{\lambda }}$ between JND in stimuli and the background stimulus is a constant, which is approximately true far beyond human senses [15,16]. Here, the similarity threshold is treated as stimuli and multi-scale clustering is performed within the above constructed Weber–Fechner observation system with parameter ${\rm{\lambda }}$.

The computation process of WFC is quite simple (Fig. 1d–f). Each ${\rm{d}}$-dimensional real-valued data point $x$ in the input dataset ${\rm{X}}$ is initially mapped to a binary code $c( x )$, using splicing/decomposable (SD) coding [17] or dimension marker (DM) coding (see Supplementary Data). Geometrically, each SD code represents a cell in a ${\rm{d}}$-dimensional mesh grid, while each DM code indicates the informative dimensions of a data point (see Supplementary Data). The selection of SD and DM coding depends on both dataset size $| {\rm{X}} |$ and data point dimension ${\rm{d}}$ (Supplementary Fig. 2). WFC makes the choice automatically to ensure its sub-quadratic time complexity with respect to $| {\rm{X}} |$ (see Supplementary Data).

In the Weber–Fechner observation system, the similarity of two points $x,\ y \in {\rm{X}}$ is defined as

(1) \begin{equation*} {\rm{sim}}\left( {x,y} \right) = \left\{ \begin{array}{@{}*{1}{c}@{}} {\frac{1}{{\delta \left( {x,y} \right)}},\quad\,\,\, {\rm SD\ coding}}\\{\frac{{H\left( {{A_{x,y}}} \right)}}{{H\left( {{O_{x,y}}} \right)}},\quad {\rm DM\ coding}} \end{array}\right., \end{equation*}

where $\delta ( {x,\ y} )$ is the Chebyshev distance between *x* and *y*, ${A_{x,y}} = \ c( x ) \wedge c( y )$, ${O_{x,y}} = \ c( x ) \vee c( y )$ and $H( \cdot )$ denotes Hamming weight (see Supplementary Data). At scale $s$, $x$ and $y$ are regarded to be similar, if ${\rm{sim}}( {x,{\rm{\ }}y} )$ is not smaller than a scale-wise threshold ${\rm{sim}}_{{\rm{min}}}^s$. For SD coding, ${\rm{sim}}( {x,{\rm{\ }}y} ) \ge {\rm{sim}}_{{\rm{min}}}^s$ means the SD codes of $x$ and $y$ represent same or neighboring cells at scale $s$. For DM coding, this indicates that $x$ and $y$ have enough common informative dimensions at scale $s$, defined by Jaccard index (see Supplementary Data).

Computing ${\rm{sim}}( {x,y} ),\ \forall x,y \in {\rm{X}}$ requires quadratic time complexity, which is a fundamental scalability bottleneck of most clustering algorithms [10]. WFC avoids this bottleneck by directly checking whether ${\rm{sim}}( {x,y} ) \ge {\rm{sim}}_{{\rm{min}}}^s,{\rm{\ }}\forall x,y \in {\rm{X}}$ in linear time with SD coding, or in sub-quadratic time with DM coding using MinHash and locality sensitive hashing [18] (see Supplementary Data).

Considering ${\rm{sim}}_{{\rm{min}}}^s$ as the background stimulus and setting its increment as JND according to the Weber–Fechner law, we have

(2) \begin{equation*} {\rm{\ sim}}_{{\rm{min}}}^{s + 1} = \left( {1 + {\rm{\lambda }}} \right) {\rm{sim}}_{{\rm{min}}}^s,\quad 1 \le s \le {{\rm{s}}_{{\rm{end}}}}. \end{equation*}

At each scale $s$, a link is added between each pair of similar codes. This constructs a graph, where each connected component (maximal connected subgraph) is regarded as a cluster (Fig. 1e and f). This process repeats from scale $s\ = \ 1$ to ${{\rm{s}}_{{\rm{end}}}}$. Finally, the clustering assignments of binary codes at all scales are mapped back to the original data, and then validated with domain-specific knowledge.

Note that in practice, WFC requires only one parameter ${\rm{\lambda }}$ to be set, which determines ${{\rm{s}}_{{\rm{end}}}}$:

(3) \begin{equation*} {{\rm{s}}_{{\rm{end}}}} = \lfloor {{\rm{lo}}{{\rm{g}}_{1 + {\rm{\lambda }}}}\left( {{\rm{si}}{{\rm{m}}_{{\rm{max}}}}/{\rm{si}}{{\rm{m}}_{{\rm{min}}}}} \right)}\rfloor , \end{equation*}

where ⌊⋅⌋ is the floor function, and ${\rm{si}}{{\rm{m}}_{{\rm{min}}}}$ and ${\rm{si}}{{\rm{m}}_{{\rm{max}}}}$ are the minimum and maximum similarity values among all data points in ${\rm{X}}$ (see details of SD and DM in the Supplementary Data). It is meaningless to set ${\rm{\ }}{{\rm{s}}_{{\rm{end}}}} > {\rm{lo}}{{\rm{g}}_{1 + {\rm{\lambda }}}}( {{\rm{si}}{{\rm{m}}_{{\rm{max}}}}/{\rm{si}}{{\rm{m}}_{{\rm{min}}}}} )$, at which all data points become dissimilar.

WFC has been implemented in Python and Apache Spark for centralized and distributed computing platforms respectively (see Methods). We validated WFC using six real datasets with up to a billion records and 2000 dimensions from distinct domains: urban taxi locations, human face images, single-cell gene expressions, computer log texts and audios. Eight popular methods were also tested for comparison, including *k*-means [11], density-based spatial clustering of applications with noise (DBSCAN) [19], hierarchical aggregation clustering (HAC) [20], affinity propagation [2], mean-shift [5], density peak [3], spectral clustering [6] and Louvain method [21] with *k* nearest neighbors [22] (*k*NN + Louvain). Experiments with small and large datasets used the centralized and distributed computing platforms respectively (Supplementary Table 1). For fair comparisons, each compared algorithm adopted different parameters across experiments to ensure its best performance (Supplementary Data Table 2).

## RESULTS AND DISCUSSION

**Figure 2.**
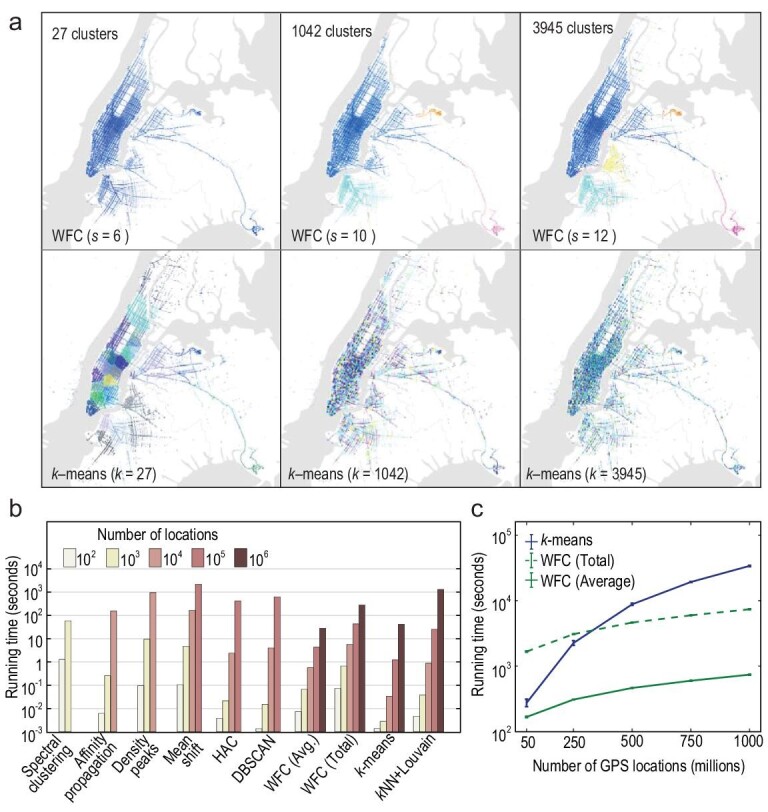
Clustering 1.1 billion taxi locations in New York City. This dataset contains 1 133 769 628 two-dimensional GPS locations (see Methods)*.* (a) Visualization of WFC and *k*-means results. The cluster numbers $k$ were set to match those identified by WFC. (b) Running time and usability of clustering algorithms with different dataset sizes using centralized computing. Different dataset sizes are obtained by slicing dataset with changing time windows (see Methods). WFC (Total) and WFC (Ave.) represent the total and average per-scale running times of WFC respectively. As dataset size increases, more and more methods fail computationally, which are not plotted. (c) Running times of WFC and *k*-means using distributed computing. The results were computed by 10 runs of each algorithm, and error bars indicate the standard error of the mean.

We performed clustering analyses to explore the spatially grouping structure within a dataset of 1 133 769 628 taxi locations in New York City (see Methods). Figure 2a illustrates results of WFC and *k*-means at three different scales. As $s$ increases, finer-grained clusters emerge, demonstrating the clustering hierarchy in the spatial distribution of taxis. For *k*-means, we set the *k* values the same as the cluster numbers identified by WFC, but its clustering results have no clear meaning. We also tested the usability and efficiency of all algorithms. As dataset size increased from 100 to 1 000 000 (using centralized computing), more methods failed to operate, except for WFC, *k*NN + Louvain and *k*-means (Fig. 2a). For more than 50 000 000 locations, only WFC and *k*-means managed to operate using distributed computing (Fig. 2b). Finally, for all 1 133 769 628 locations, WFC detected hierarchical clusters over 25 scales, using only 0.1 running time of *k*-means. Multi-scale clustering provided by WFC could empower various applications of urban-scale planning and decision making [23].

**Figure 3.**
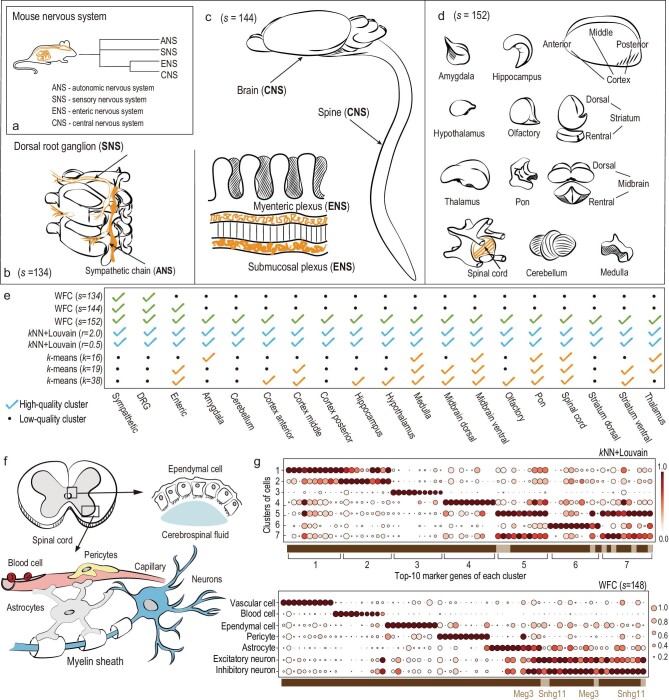
Clustering 507 286 single cells of the mouse nervous system and 37 221 spinal cord cells. (a) Illustration of anatomical regions of the mouse nervous system, and a high-level clustering hierarchy established by WFC. (b–d) Nervous tissues (clusters) identified by WFC over three different scales. (e) Results of clustering all nervous single cells. High-quality clusters are with at least 100 cells and 0.9 purity score [24]. (f) Illustration of the main cell types of the spinal cord. (g) Clustering spinal cord cells using WFC and *k*NN + Louvain. Top-10 marker genes of each cluster (at least 100 cells) are plotted as circles. Color darkness represents the mean expression of this gene (min-max normalized), and circle size represents the fraction of cells expressing this gene within the corresponding cluster. A marker gene may belong to a single cluster or multiple clusters, represented by dark and light brown grids respectively in the horizontal axis. WFC detects seven clusters identifying specific cell types. *k*NN + Louvain finds 30 clusters (Supplementary Fig. 6), and the best seven are plotted here.

Scalable hierarchical clustering is central to identifying cell types and building cell atlases based on scRNA-seq data [8,9]. We used clustering methods to detect hierarchical anatomical regions of the mouse nervous system (Fig. 3a) based on an scRNA-seq dataset of 507 286 cells with 2000-dimensional informative gene expressions of the mouse nervous system [8] (see Methods). This dataset was organized and labeled using *k*NN + Louvain and polished with domain knowledge. Only WFC, *k*NN + Louvain and *k*-means managed to analyze this dataset computationally. Figure 3b and d illustrate the hierarchy of tissues (clusters) identified by WFC. *k*NN + Louvain detected all fine-grained tissues, but failed to establish the clustering hierarchy by changing the resolution setting *r* from 0.5 to 2.0 (Fig. 3e and Supplementary Fig. 4). *k*-means performed poorly in both accuracy and clustering hierarchy for $k\ = {\rm{\ }}10,{\rm{\ }}11,{\rm{\ }} .\,.\,.,{\rm{\ }}38$ (Supplementary Fig. 3). We next applied clustering to identify cell types of the spinal cord (Fig. 3f). WFC detected seven clusters with obvious marker genes (Fig. 3g). Here, identified clusters are well separated according to the marker genes, and each of them identifies a known cell type of the spinal cord (see Methods). *k*NN + Louvain detected 30 clusters with its optimal setting *r *= 1 [8], and the best seven are illustrated in Fig. 3g, in which the marker genes are much less representative. Complete results are illustrated in the Supplementary Data. This demonstrates that WFC would be a better alternative to the popular Louvain method for massive scRNA-seq data clustering.

**Figure 4.**
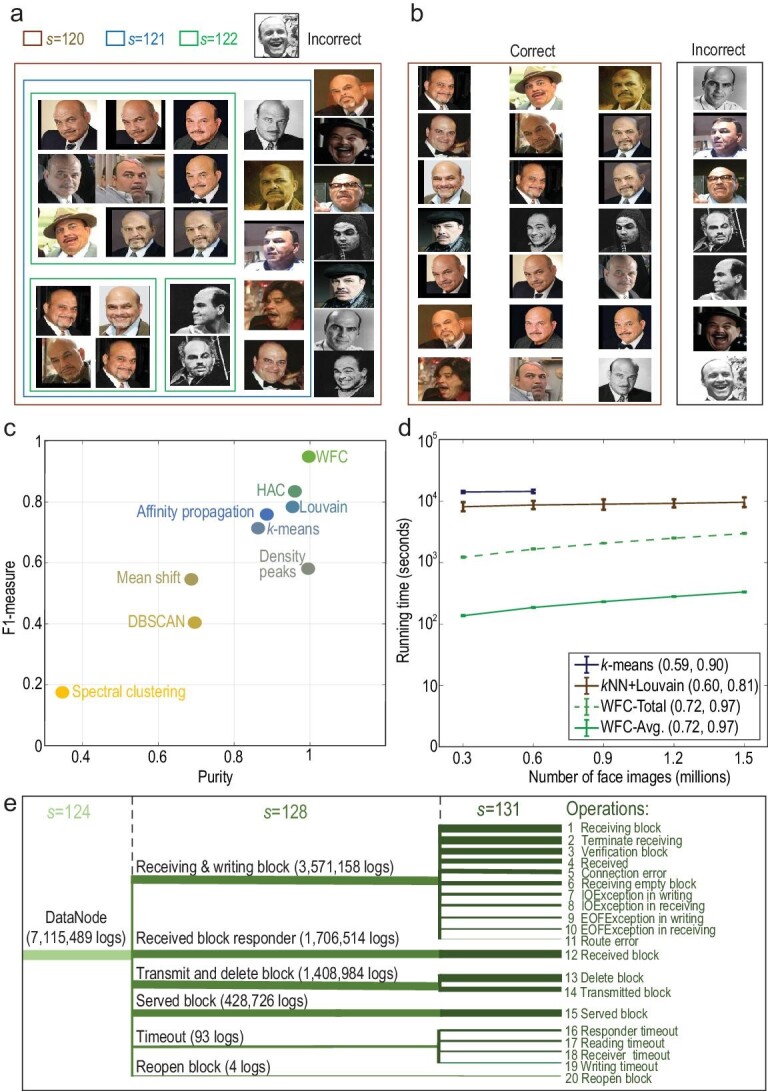
Clustering 1.5 million face images and 11 million HDFS log texts. (a and b) Results of 28 photos labeled ‘Jon Polito’ in clustering the first 500 images by using (a) WFC and (b) *k*-means respectively. There are 24 labels of the first 500 images. By setting $k\ = {\rm{\ }}24$, *k*-means achieves its highest F1-measure score (0.809), resulting in only 21 correctly classified images. WFC identified a cluster with 27 correct images at $s\ = {\rm{\ }}120$ in a fully unsupervised way. Finer clusters with stronger similarities were also detected at the next two scales. (c) Validation scores for clustering the first 500 images. (d) Running times and evaluation scores (F1-measure, purity) of WFC, *k*-means and *k*NN-Louvain using distributed computing. Each result in (c and d) was computed by running each experiment 10 times, and error bars indicate the standard error of the mean. (e) Multi-scale clusters of HDFS logs detected by WFC, representing meaningful HDFS operations at different hierarchical levels. The width of each line is proportional to the logarithm of corresponding cluster size.

We next applied clustering algorithms to a dataset of 307 784 high-quality face images (512-dimensional feature vector) of 10 567 people (see Methods). All methods managed to cluster the first 500 images (24 people), while WFC achieved the highest scores of F1-measure and purity (Fig. 4c), two representative clustering evaluation metrics [24]. In addition, WFC managed to establish the clustering hierarchy of face photos (Fig. 4a and b). To test efficiency, we copied all 307 784 images up to five times (1 538 920 images). *k*-means failed to cluster more than 615 568 images, while WFC ran nearly 10 times faster than *k*NN + Louvain (Fig. 4b). The effectiveness and efficiency of multi-scale face clustering would benefit various applications in social media and public security.

Analysis of log texts is essential for understanding the operational behaviors of computing systems serving us every day, from smartphones to the cloud. To test WFC in this context, we considered 11 197 954 log texts of the Hadoop Distributed File System (HDFS), a popular software for large-scale data storage. Each log was represented as a 600-dimensional feature vector (see Methods). Except for WFC and *k*-means, all methods failed in clustering more than 10 000 000 logs, and WFC achieved significantly higher validation scores than others (Supplementary Figs 9 and 10). A meaningful hierarchy of HDFS operations was also successfully established by WFC (Fig. 4e), showing the potential of WFC in unsupervised analysis of complex software behaviors.

WFC also identified multi-scale meaningful clusters in an audio dataset with 22 176 10-second audio segments (see Methods). Different music genre styles (e.g. opera, Indian music and Latin American music) were detected among all audio segments at $s\ = \ 95$. Then, different instrument types (e.g. guitars, bowed strings and keyboards) were further identified at $s\ = \ 96$ (Supplementary Fig. 11). This could be useful for various data-driven music applications.

**Figure 5.**
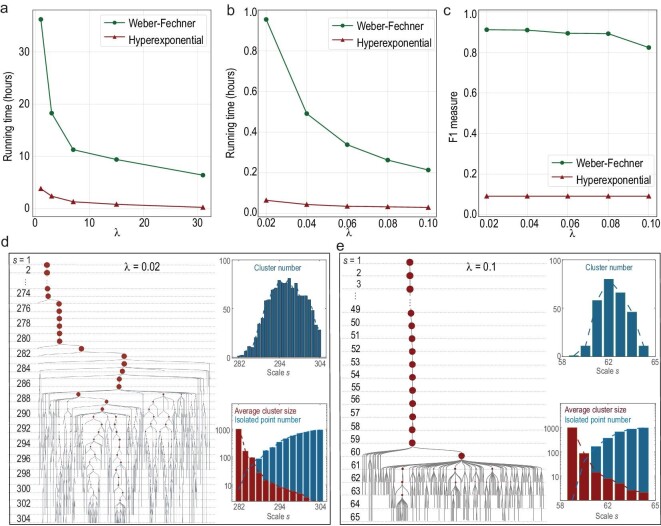
The impact of scale updating on efficiency, effectiveness and clustering hierarchy. (a and b) Running times of WFC with different ${\rm{\lambda }}$ settings and scale-updating functions for the first 50 million taxi locations in the NYC dataset and the first 1000 images in the CASIA-webface dataset respectively, where the hyper-exponential function is ${\rm{\ sim}}_{{\rm{min}}}^s = {( {1 + {\rm{\lambda }}} )^{{{( {s - 1} )}^2}}}\ {\rm{si}}{{\rm{m}}_{{\rm{min}}}}$. (c) F1-measure of WFC with different ${\rm{\lambda }}$ settings and scale-updating functions for the 1000 images. (d and e) Visualization of clustering hierarchy and statistical results for the 1000 images with different ${\rm{\lambda }}$ settings. The radius of each brown circle (cluster) is proportional to the square root of the corresponding cluster size. For a clear visualization, each isolated data point $x$ and the link to its parent cluster (the cluster containing $x$ in the last scale) are not plotted.

Finally, we studied the impacts of ${\rm{\lambda }}$, the only parameter of WFC, on efficiency, effectiveness and clustering hierarchy. We can see that a smaller ${\rm{\lambda }}$ results in a higher F1-measure score (Fig. 5c) but a longer running time (Fig. 5a and b), since more hierarchical layers are conducted and less meaningful clusters are missing between scales (Fig. 5d and e, Supplementary Figs 12 and 13). Computationally, we can also use other functions besides the exponential function (Weber–Fechner law) to update scales, although they may have no psychological meaning. It can be seen that the hyper-exponential updating policy is much faster, which is highly desired for massive data clustering (Fig. 5a and b), but it achieves very poor F1-measure scores (Fig. 5c) due to the large number of meaningful clusters missed between aggressively updated scales. In contrast, the Weber–Fechner law can achieve both reasonable running time and efficiency simultaneously.

## CONCLUSION

Psychological principles have inspired several solutions to computer science and artificial intelligence problems, such as the Weber–Fechner law in signal processing [25], Gestalt laws for clustering [26], Fitts’s law in the human–computer interface [27] and the Yerkes–Dodson law for affective computing [28]. Our work demonstrates the advantages of applying multi-scale cognitive principles to discover complex grouping structures hierarchically hidden in massive datasets. To our knowledge, WFC is the first method that applies the multi-scale cognition process with the Weber–Fechner law for massive data clustering. This simple, fast, effective and interpretable unsupervised learning method could empower advanced large-scale data analysis in various disciplines.

## METHODS

### Implementation

We provide Python and Apache Spark [29] implementations for centralized (stand-alone) and distributed computing respectively. Codes and data used in this paper are both available at github.com [30]. WFC takes original dataset data and parameter ${\rm{\lambda }}$ as input. See Supplementary Data for more detailed experiment settings and parameters.

### Definition of purity and F-measure

Purity and F-measure are popular validation measures for flat clustering [24]. For a dataset ${\rm{X}}$ partitioned by a set of clusters $C$ and a set of labeled classes$\ L$, the global purity score can be computed as

$$\begin{equation*}
{\rm{Purity}}\left( C \right) = \frac{1}{{\left| {\rm{X}} \right|}}\ \mathop \sum \limits_{c \in C} \mathop {\max }\limits_{l \in L} \left| {c \cap l} \right|,
\end{equation*}$$

where ${\rm{ma}}{{\rm{x}}_{l \in L}}| {c \cap l} |$ is the purity score of a specific cluster $c \in C$. Consider a cluster $c$ and a labeled class $l$, denote recall ${R_{c,l}} = | {c \cap l} |/| l |$ and precision ${P_{c,l}} = | {c \cap l} |/| c |$, their F1-measure is

$$\begin{equation*}
{\rm{F}}1 \left( {c,l} \right) = \frac{{2{R_{c,l}}{P_{c,l}}}}{{{R_{c,l}} + {P_{c,l}}}}.
\end{equation*}$$

The global F1-measure score is computed as

$$\begin{equation*}
{\rm{F}}1{\rm{ {-} measure }}\left( C \right) = \frac{1}{{\left| L \right|}}\ \mathop \sum \limits_{l \in L} \mathop {\max }\limits_{c \in C} \left( {{\rm{F}}1\left( {c,l} \right)} \right).
\end{equation*}$$

### Clustering taxi locations in New York City

Taxi locations used in this experiment are based on records of yellow taxis from the New York City (NYC) Taxi & Limousine commission [31]. Each record includes time tags, latitude–longitude locations and taxi trip information. We only use the start and end locations of all taxi trips falling in the area (N 40.5°–40.9°, W 73.6°–74.2°). There are a total of 1 157 184 863 records during exactly seven years from 1 January 2009 (00 : 00 : 00) to 31 December 2015 (23 : 11 : 59). To reduce outliers, records with extremely small densities were filtered [30], i.e. those with densities smaller than 90 locations per 0.01° latitude–longitude area. Finally, we obtained 1 133 769 628 locations for clustering. SD coding was used for this massive and two-dimensional dataset. By setting $\lambda {\rm{\ }} = \ 1$, we have ${{\rm{s}}_{{\rm{end}}}} = \ 25$. From scales 1–5, there is one cluster and isolated points, and validated clustering results emerge at scale 6.

### Clustering single-cell gene expressions of mouse nervous systems

This experiment is based on the level 1 subset of the Mouse Brain Atlas dataset [32], a collection of 507 286 single cells represented as 27 998-dimensional vectors of gene expressions. Each cell has a label of 19 tissues in the mouse nervous system. We selected the top 2000 informative genes with the highest variances [8,33] to represent each cell for clustering. By treating the labels as ground truth, we first applied clustering algorithms to detect 19 tissues of all 507 286 single cells (Fig. 3a–e, Supplementary Fig. 5). Then we used clustering algorithms for 37 221 spinal cord cells to identify cell types. Marker genes shown in Fig. 3f–g and in Supplementary Figs are illustrated using SCANPY 1.3 [34]*.* More detailed specific parameter settings of all clustering algorithms are provided in the Supplementary Data.

### Clustering face photos

CASIA-webface dataset [30] is a collection of 494 414 facial images of 10 575 people (labels), and the number of images for each person ranges from 2 to 817. Since the original dataset contains many images with low resolution and undetectable faces, filtering is required for data pre-processing. We adopted a commonly used face detector [35], and set the minimal threshold of the returned quality score as 1. After filtering, we obtained 307 784 high-quality facial images of 10 567 people. Then, a 512-dimensional feature vector of each image was extracted by the deep learning model LResNet34E-IR [36]. Minhash-LSH is used for clustering this high-dimensional dataset. Detailed parameter settings of all algorithms are summarized in the Supplementary Data.

### Clustering logs of the HDFS

This experiment is based on the SOSP 2009 dataset [37] containing 11 197 705 logs of the HDFS [38] in a private cloud. Each log consists of four segments of information: time tag, log type (Information, Warning, Error), source name (i.e. from which component the log is generated) and the operation details. The dataset has 25 labeled samples as the ground truth listed in Supplementary Table 8. Each log was transformed into a 600-dimensional feature vector using word to vector (Word2Vec) embedding [39]. Parameter settings of both Word2Vec and all tested algorithms are summarized in the Supplementary Data.

### Clustering audios

This experiment is based on the 22 176 audios of ‘balanced_train_segments’ in AudioSet [40]. All audios have the same length of 10 seconds and were converted into 128-dimensional feature vectors. This dataset has 527 labels in total, and each audio has at least 59 labels. The ${\rm{\lambda \ }}$of the algorithm is set as 0.05. The audio contents vary widely and mislabeling exists. We focused on ‘Music’ and all instrument-related labels [41] due to their high accuracy, and the large number of corresponding audios [42]*.* These labels can provide a validated ground truth for evaluating the clusters detected by WFC.

## DATA AVAILABILITY

The open-source code of WFC is available on GitHub (https://github.com/IoTDATALab/WFC). The persistent specific version of WFC is available at Zenodo [30]. The datasets associated with this work and the supporting data for figures are available at https://doi.org/10.5281/zenodo.4297399 [30].

## Supplementary Material

nwab183_Supplemental_File
