# Supplementary material for: Massive data clustering by multi-scale psychological observations
Source: Natl Sci Rev. 2021 Oct 8;9(2):nwab183. doi: 10.1093/nsr/nwab183 (PMC8889001; doi:10.1093/nsr/nwab183)
Supplement: nwab183_Supplemental_File [file nwab183_supplemental_file.docx]

Supplementary Information for

Massive Data Clustering by Multi-scale Psychological Observations

Shusen Yang1,2^*†^, Liwen Zhang1^†^, Chen Xu3^†^, Hanqiao Yu1, Jianqing Fan4^*^ & Zongben Xu1^*^

1 *National Engineering Laboratory of Big Data Analytics, Xi’an Jiaotong University, China*

2 *Pazhou Laboratory, Guangzhou, China*

3 *Department of Mathematics and Statistics, University of Ottawa, Canada*

4 *Center for Statistics and Machine Learning, Princeton University, USA*

† *These authors contributed equally to this work.*

*^*^* *Corresponding authors. Emails:* [*shusenyang@mail.xjtu.edu.cn*](mailto:shusenyang@mail.xjtu.edu.cn)*;* [*jqfan@princeton.edu*](mailto:jqfan@princeton.edu)*;* [*zbxu@mail.xjtu.edu.cn*](mailto:zbxu@mail.xjtu.edu.cn)

**Splicing/Decomposable (SD) Coding for low-dimensional data clustering**

The SD coding method developed in the work^1^ is used to map each data point x in original dataset $X$ into a binary code $c^{s}(x)$ at each scale s in the Weber-Fechner observation system for fast computation. SD codes represent data with different precision levels using simple binary operations, which can efficiently support the identification of similar pairs over multiple scales. Given a d-dimensional dataset $X\subset\mathbb{R}^{d}$, the axis-aligned minimum bounding box of X is divided into a mesh grid of 2dh cells (i.e., each cell is a d-dimensional hyper-rectangular), by halving h times over each dimension. We denote the maximum and minimum Chebyshev distances between all data points in $X$ as

$\delta_{\max}=\max_{x,y\in X} \left\| x-y \right\|_{\infty}$

$\delta_{\min}=\min_{x,y\in X, \left\| x-y \right\|_{\infty}>0} \left\| x-y \right\|_{\infty}$

where $\left\| \right\|_{\infty}$represents the infinite norm. Here, all data points within a given cell are represented by the same binary SD code of this cell. For given *h*, an SD-code $c^{h}$ consists of a sequence of *h* d-bit binary precision-specific sub-codes,$pc^{1}$ to $pc^{h}$, by halving each dimension h times:

$c^{h}=\left[ pc^{1} \right]\left[ pc^{2} \right]\cdots\left[ pc^{h} \right]$

$=\left[ {bit}_{1}^{1},{bit}_{2}^{1}, \cdots{bit}_{d}^{1} \right]\left[ {bit}_{1}^{2},{bit}_{2}^{2},\cdots,{bit}_{d}^{2} \right]\cdots\left[ {bit}_{1}^{h},{bit}_{2}^{h},\cdots,{bit}_{d}^{h} \right]$

Here, each bit ${bit}_{i}^{j}$ indicates one of the two coordinate intervals of the $j$th halving at the $i$th dimension. Obviously, *h* decides the precision of SD code. Let $\delta_{h}=\delta_{\max}/2^{h}$ be the largest length of all cell edges, *h* can be increased until $\delta_{h}<\delta_{\min}$. In this case, SD code achieves no precision loss, because each cell contains at most one data point. Therefore, SD codes can naturally support multi-scale observations by increasing *h* from 1 to $\left\lceil\log_{2}(\delta_{\max}/\delta_{\min}) \right\rceil$, where $\left\lceil\right\rceil$ is the roof function. For WFC with parameter $\lambda$ at each scale *s*, the corresponding halving function is computed as:

$$h(s)= \left[ \log_{2}\left( 1+\lambda\right)^{s} \right], s∊[1, s_{\mathrm{end}} ]$$

For each dimension$1\leq i\leq d$of a given SD code $c^{h(s)}$, define its dimension-specific sub-codes as

${dc}_{i}^{h(s)}=\left\langle{bit}_{i}^{1},{bit}_{i}^{2},\cdots{bit}_{i}^{h(s)} \right\rangle$, $i∊[1, d ]$

**Definition 1 [Neighboring cells].** Two different SD codes (cells) $c^{h(s)}\left( x \right)$ and $c^{h(s)}\left( y \right)$ are defined as neighboring cells, if the maximal difference between them at all dimensions is equal to 1, i.e., $\max_{1\leq i\leq d} |{dc}_{i}^{h\left( s \right)}\left( x \right)-{dc}_{i}^{h\left( s \right)}\left( y \right)|=1$.

Geometrically, two neighboring cells share at least one vertex in the d-dimensional mesh grid. At scale s, two data points $x,y\in X$ are regarded as similar if their corresponding SD codes $c^{h(s)}\left( x \right)$ and$c^{h(s)}\left( y \right)$ are same or neighboring cells. Therefore, we have

$$\mathrm{sim}_{\min}^{s}=\frac{1}{\delta_{h\left( s \right)}}\leq\frac{1}{\delta\left( x,y \right)}=\mathrm{sim}\left( x,y \right), x,y\in X, s∊\left[ 1, s_{\mathrm{end}} \right]$$

where $\delta\left( x,y \right)$ is the Chebyshev distance between x and $y$. **It is also obvious that the maximum and minimum similarities are:** $\mathbf{sim}_{\mathbf{max}}\mathbf{=1/}\boldsymbol{\delta}_{\mathbf{min}}$ **and** $\mathbf{sim}_{\mathbf{min}}\mathbf{=1/}\boldsymbol{\delta}_{\mathbf{max}}$**.**

With SD coding, WFC can directly identify all neighboring cells of each given $c^{h(s)}\left( x \right), x\in X$ using just binary subtraction and addition, i.e., *O*(1) time complexity with respect to |X|. Supplementary Fig. 1 shows an example of a two-dimensional case. Since each d-dimensional cell has totally $3^{d}-1$ neighboring cells (each coordinate can go up or down one cell or no change) to be identified, SD coding method is quite fast for massive but low-dimensional datasets but is not suitable for high dimension cases.

**Dimension Marker (DM) coding for high-dimensional data clustering**

For a given d-dimensional dataset $X\subseteq\mathbb{R}^{d}$, each data point is represented as a positive real-valued feature vector $x=(x_{1}$, $x_{2}$, …, $x_{d}$). The DM code of $x$ is a d-length binary code that remains the same over all observation scales:

$$c\left( x \right)=[{bit}_{1},{bit}_{2}, \cdots{bit}_{d}]$$

The $i$ th bit of a DM Code is computed as

$${bit}_{i}=\left\{ \begin{aligned} 1, &x_{i}>{Tr}_{i} \\ 0, &\mathrm{otherwise} \end{aligned} \right.$$

where ${Tr}_{i}$ is a cut off threshold for dimension $i$: for sparse high-dimensional data (e.g., mouse brain single cells), ${Tr}_{i}$ can be simply set as zero. For dense high-dimensional data, this can be set according to different criteria. For example, in the experiments of face images and computer logs, ${Tr}_{i}$ is set as half of the maximum value for each dimension $i$: ${Tr}_{i}=\max_{x\in X}(x_{i})/2$.

Let S(x) be a set of corresponding key-value pairs ($i$, ${bit}_{i}$), $1\leq i\leq d$. The similarity between two data points x and y is defined as the Jaccard index between $S\left( x \right)$ and $S(y)$,

$$\mathrm{sim}\left( x,y \right)=\frac{|S(x)\cap S(y)|}{|S\left( x \right)\cup S\left( y \right)|}=\frac{H(c\left( x \right)\wedge c\left( y \right))}{H(c\left( x \right)\vee c\left( y \right))}$$

which measures the extent of overlaps in the dimensions of $x$ and $y$. Here,$H(\cdot)$ denotes Hamming weight, i.e., the total number of non-zero bits of a given binary code. For WFC with given $\lambda$ at scale s, the similarity threshold $\mathrm{sim}^{\boldsymbol{s}}$ is updated as

$\mathrm{sim}_{\min}^{s+1}=(1+\lambda)\mathrm{sim}_{\min}^{s}$**,**$s∊[1\boldsymbol{,}s_{\mathrm{end}} \boldsymbol{]}$

where $\mathrm{sim}_{\min}^{1}=1/d$ is the minimal Jaccard index when two data points have one common dimension and the union of them covers all d dimensions. **Therefore, we have the minimum and maximum similarity values:** $\mathbf{sim}_{\mathbf{min}}\mathbf{=1/d}$ **and** $\mathbf{sim}_{\mathbf{max}}\mathbf{=1}$**.**

Minhash and Locality Sensitive Hashing (Minhash-LSH)^2^ are fundamental similarity indexing tools, which can quickly identify similar sets defined by Jaccard index threshold in sub-linear time^3^. Minhash-LSH has been implemented in many popular programming languages and platforms of data science and machine learning (e.g., Python, Spark). WFC sets $\mathrm{sim}_{\min}^{s}$ as the input parameter of Minhash-LSH at each scale s, and the set of all DM codes similar to a given DM code $c^{s}\left( x \right)$can be obtained in sub-linear time.

**Remarks**

**Remark 1. Selection between SD coding and Minhash-LSH.** In general, WFC uses SD coding and MinHash-LSH for low-dimensional and high-dimensional datasets respectively. Supplementary Fig. 6 illustrates the selection criteria between two methods for an input $d$-dimensional dataset X with desired neighbour identification time complexity bounds. In practice, if the point ($d$, $|X|$) falls in the area of the desired complexity bound (e.g., square root time), WFC adopts SD coding; otherwise, Minhash-LSH. WFC adopts the square root bound as the default selection criterion in both Python and Spark implementations.

**Remark 2. Sub-quadratic complexity of WFC with respect to dataset size.** WFC requires three computation steps: map input data to binary codes $O(\mathrm{coding})$, multi-scale clustering $O(\mathrm{clustering})$, and decode clustering results to the original data $O(\mathrm{decode})$. We further define $O\left( \mathrm{Similarity} \right)$ and $O(Connected\_component)$ as the time complexities of identifying all similar pairs of binary codes and computing all connected components (maximal connected subgraph) of a given graph respectively. We have:

$O\left( \mathrm{WFC} \right)=O\left( \mathrm{coding} \right)+O\left( \mathrm{clustering} \right)+O(\mathrm{decoding})$

$=_{(a)}O\left( |X| \right)+O\left( \mathrm{clustering} \right)+O\left( |X| \right)$

$=_{(b)}O\left( |X| \right)+s_{\mathrm{end}}(\left| X \right|O\left( \mathrm{Similarity} \right)+O(Connected\_component))$

$=_{(c)}O\left( |X| \right)+\left\lfloor\log_{1+\lambda}(\mathrm{sim}_{\max}/\mathrm{sim}_{\min}) \right\rfloor(\left| X \right|O\left( \mathrm{Similarity} \right)+O(Connected\_component))$

$=_{(d)}O\left( |X| \right)+\left| X \right|O\left( \mathrm{Similarity} \right)+O(Connected\_component)$

$=_{(e)}O\left( \left| X \right| \right)+\left| X \right|o\left( \left| X \right| \right)+O\left( \left| X \right| \right)$

$=o({|X|}^{2}$)

Here, equality (a) holds because both mapping and reverse mapping require linear time. Equalities (b)-(d) demonstrate that $S_{\mathrm{end}}$ is independent to $|X|$. Equality (e) holds because that neighbor identification is sub-linear for both SD Coding and DM coding, and all connected components can be obtained by using breadth-first search in linear time.

**Remark 3: Discussions on convergence and robustness.** The convergence of WFC is quite straightforward. WFC only has one parameter λ without an iteration parameter (algorithms like *k*-means). Within sub-quadratic time (with respect to the dataset size |X|), WFC will definitely stop and a hierarchy of clustering results can be obtained. In addition, as a universal massive data clustering algorithm, WFC does not rely on any model with assumptions such as data and noise distributions. Extensive experiments also validate the robustness of WFC across various real clustering tasks with distinct datasets.

**Remark 4: Producing flat clustering assignments by detected hierarchal clusters.** The additional dimension of scale enables the representation of hierarchically overlapping clusters. Multiple flat clustering assignments can be produced though the combination of clusters detected at different scales. Taking Fig.1 for example, we can obtain a flat clustering assignment that includes all nine clusters (red) at scale four and two new clusters produced by removing all data points in red clusters at scale four from their ancestor clusters (yellow and blue) at scale two.

# Remark 5: Recognizing clusters beyond connected components at each scale. WFC defines connected components as a cluster, because they reflect the fundamental grouping structure in graphs and can be identified in linear time with breadth-first search. More complex topological structures could also be adopted to define clusters. For instance, one can define communities as clusters and use Louvain method^4^ to detect clusters at each scale in the Weber-Fechner observation system, but this will result in higher computational complexity and more parameters to tune in practice.

# Remark 6: Updating Scales beyond Weber-Fechner law. For Psychological interpretability, WFC adopts Weber-Fechner law (an exponential scale updating function) to simulate the multi-scale observation process of real-world entities of humans. From the pure computational aspect, exponential function can balance the tradeoff between efficiency and effectiveness by simply changing parameter λ, while it is also straightforward to use other functions for scale updating in WFC. Furthermore, additional methods such as dynamically changing λ or function formats over scales can also be easily adopted to our original WFC algorithm. However, this paper focuses on the original WFC to achieve the four essential objectives (Interpretability, High scalability, Universality, and User friendliness) simultaneously. Possible computational extensions are remained for our future work. The publicly available Python and Spark codes of WFC can also greatly facilitate extensions for the research community and users.

# Further Details of the Experiments

**Computing Platforms and Experiment setting.**

As mentioned in the main text, we use centralized computing platforms for small datasets and distributed ones for large datasets. Detailed experiment settings are listed in Supplementary Tables 3-7.

**Clustering taxi locations in New York City (NYC).**

Parameter settings of all algorithms are summarized in Supplementary Table 3. By setting $=1$, we have $s_{\mathrm{end}}=25$. From scales 1-5, there are one cluster and isolated points, and validated clustering results emerge at scale six.

**Clustering single-cell gene expressions of mouse nervous systems.**

Figs 3a-3e in the main text illustrate the clustering of 507,286 scRNA-seq single cells data of the mouse nervous system, which is more explicitly presented in Supplementary Figure 3.

Supplementary Figures 4-7 are the results of detecting cell types of spinal cord by different clustering algorithms. Parameter settings of all clustering algorithms of single cells scRNA-seq clustering are listed in Supplementary Table 4.

**Clustering face photos.**

Parameter settings of face photo clustering experiment of all algorithms are summarized in Supplementary Table 5.

**Clustering logs of Hadoop Distributed File System (HDFS).**

Supplementary Table 8 exemplifies the segments of information that make up the dataset of HDFS logs. The 25 ground truths mentioned in the main text of the dataset are listed in Supplementary Table 9. Parameter settings of both Word2Vec and all tested algorithms for HDFS logs clustering are summarized in Supplementary Table 6.

Supplementary Fig. 10 compares the results of HDFS logs clustering by WFC with those by other clustering algorithms. Supplementary Figs 9 and 10b show that WFC achieves significantly higher validation score than other algorithms.

# References

1. Xu, Z.-B., Leung, K.-S., Liang, Y. & Leung, Y. Efficiency speed-up strategies for evolutionary computation: fundamentals and fast-GAs. *Appl. Math Comput.* **142**, 341–388 (2003).

2. Broder, A. Z. On the Resemblance and Containment of Documents. in *In Compression and Complexity of Sequences (SEQUENCES’97* 21–29 (IEEE Computer Society, 1997).

3. Andoni, A. & Indyk, P. Near-optimal hashing algorithms for approximate nearest neighbor in high dimensions. *Commun. ACM* **51**, 117–122 (2008).

4. Blondel, V. D., Guillaume, J.-L., Lambiotte, R. & Lefebvre, E. Fast unfolding of communities in large networks. *J. Stat. Mech.* **2008**, P10008 (2008).

5. Manning, C. D., Raghavan, P. & Schütze, H. *Introduction to Information Retrieval*. (Cambridge University Press, 2008).


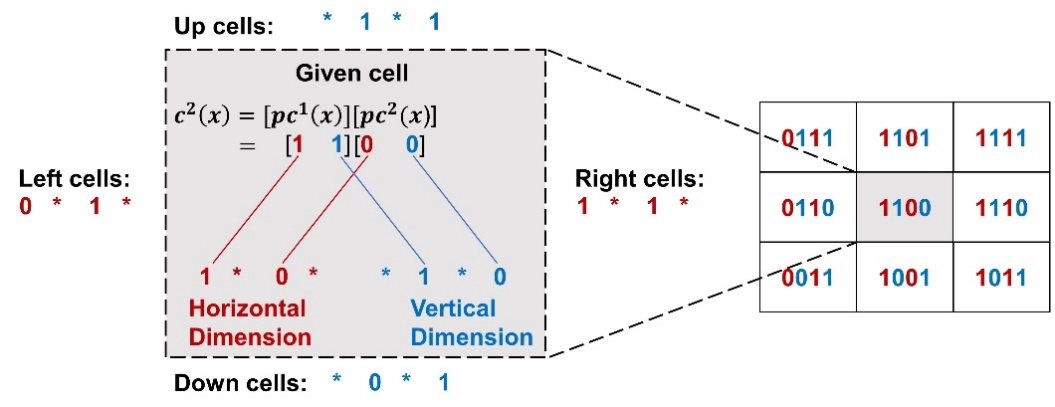


**Supplementary Figure 1 Illustration of neighboring cell identification for a two-dimensional SD code with *h*=2.** The given SD code [1,1,0,0] consists of two precision-specific sub-codes [1,1] and [0,0]. This gives the horizontal and vertical coordinates of the corresponding cells, i.e., both of their dimension-specific sub-codes are <1,0>, corresponding to the third interval of each coordinate. Therefore, the coordinates of all eight neighboring cells, among 16 cells and their SD codes can be directly computed: up (adding 1 to the blue code) or down (subtracting 1 from the blue code), left (subtracting 1 from the red code), right (adding 1 to the red code), and 4 diagonal cells (e.g., add 1 to blue and add 1 to red, subtract 1 from blue and add 1 to red).


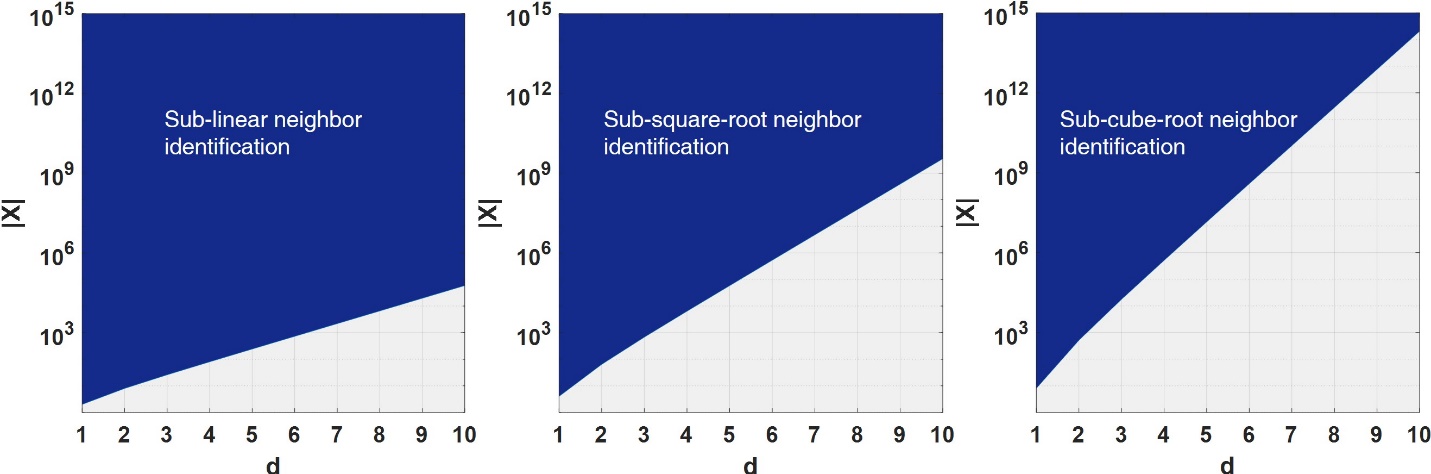


**Supplementary Figure 2** Time complexity of neighbor identification with SD coding for an input $d$-dimensional dataset $X$. The point $\left( d,\left| X \right| \right)$ falls in the blue area means that the corresponding time complexity of neighbor identification can be achieved by using SD coding. In practice, if the point ($d$, $|X|$) falls in the area of the desired complexity bound (e.g., square root time), WFC adopts SD coding; otherwise, Minhash-LSH. WFC adopts the square root bound as the default selection criterion in both Python and Spark implementations.

**
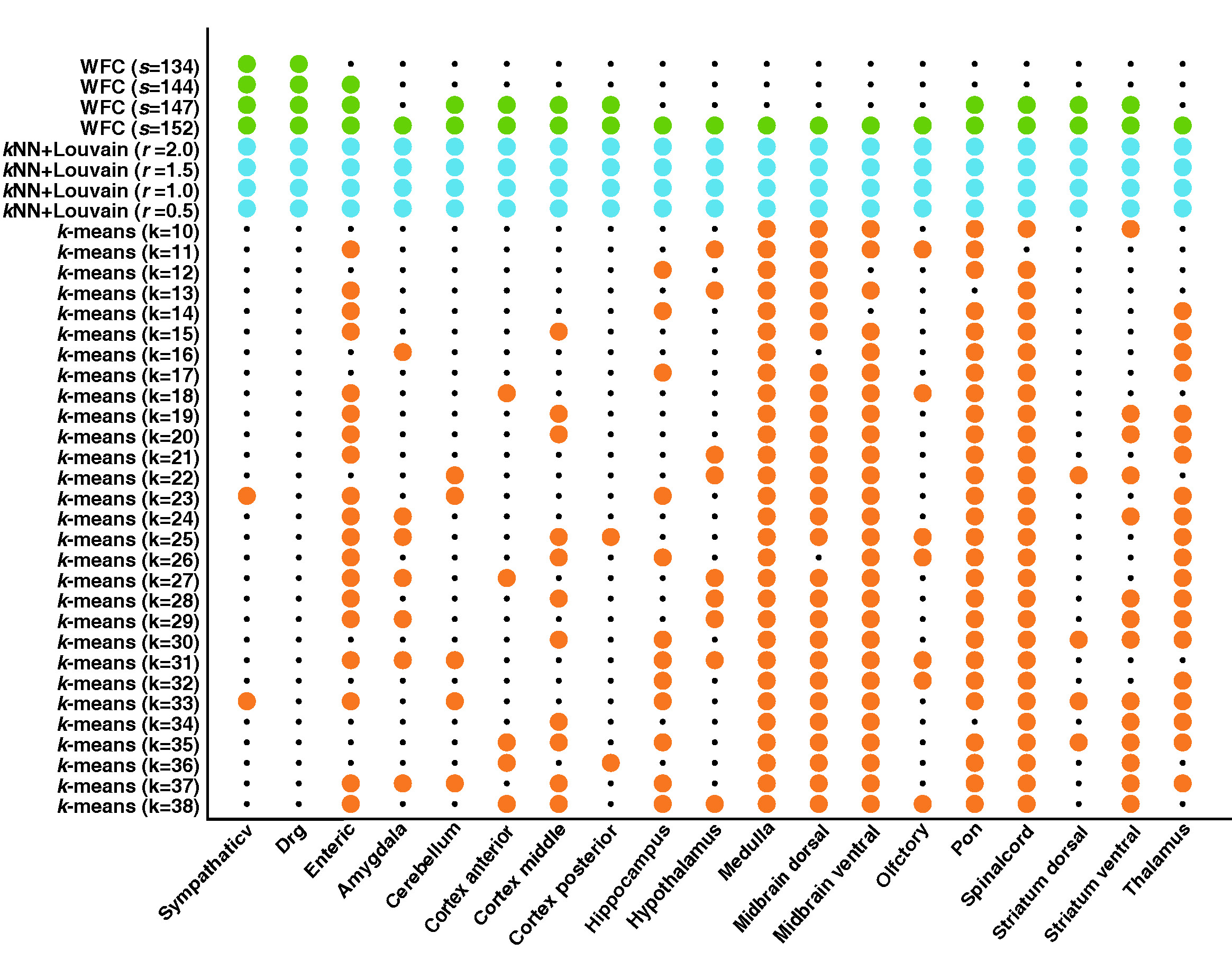
**

**Supplementary Figure 3. Detection tissues of the mouse nervous system from 507,286 scRNA-seq single cells data.** Each circle represents a high-quality cluster, which contains at least 100 cells and achieves more than 0.9 purity score^5^. Dots indicate low-quality clusters that do not meet the condition.


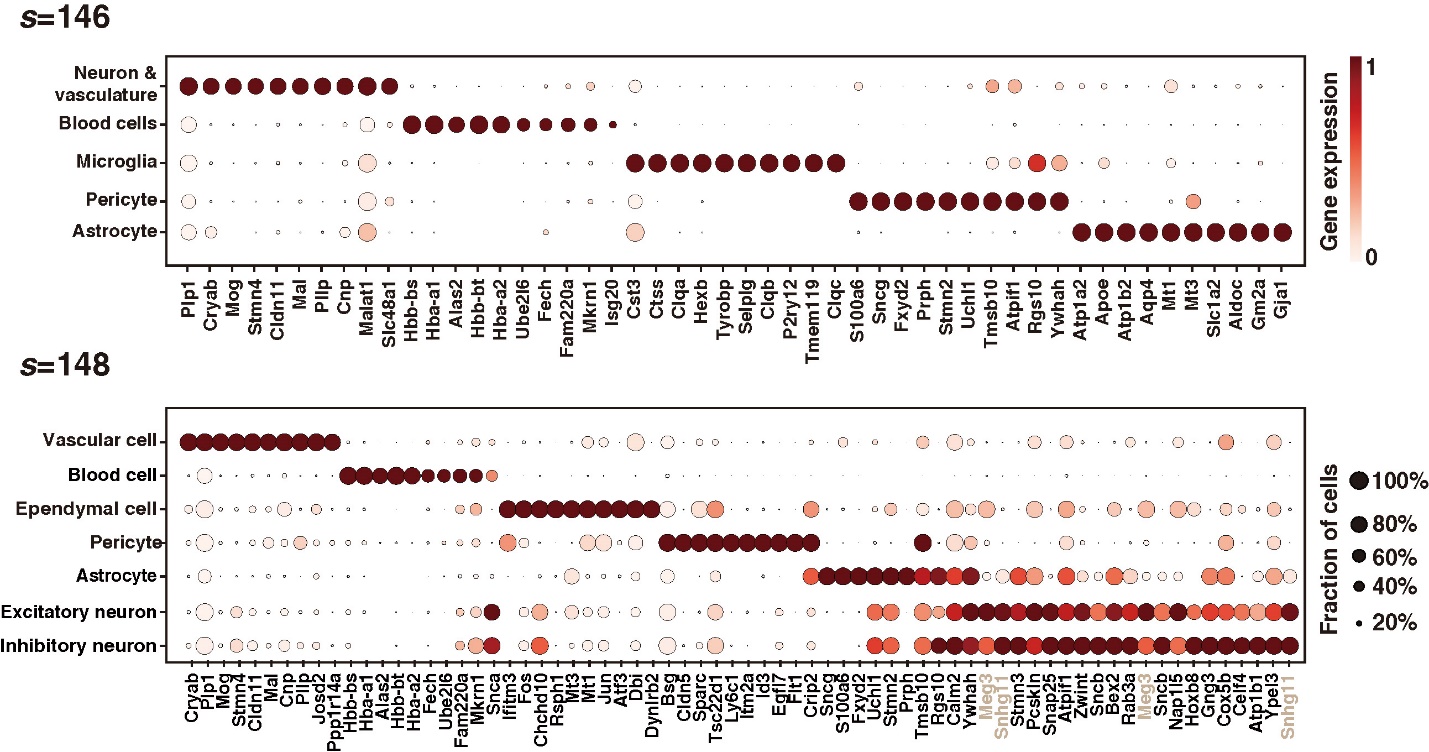


Supplementary Figure 4. Cell types of spinal cord detected by WFC. Top 10 marker genes of each cluster (at least 100 cells) are plotted as circles. Color darkness represents the mean expression of this gene (min-max normalized), and circle size represents the fraction of cells expressing this gene within the corresponding cluster. Genes belonging to a single cluster or multiple clusters are represented by black and light brown colors.

_
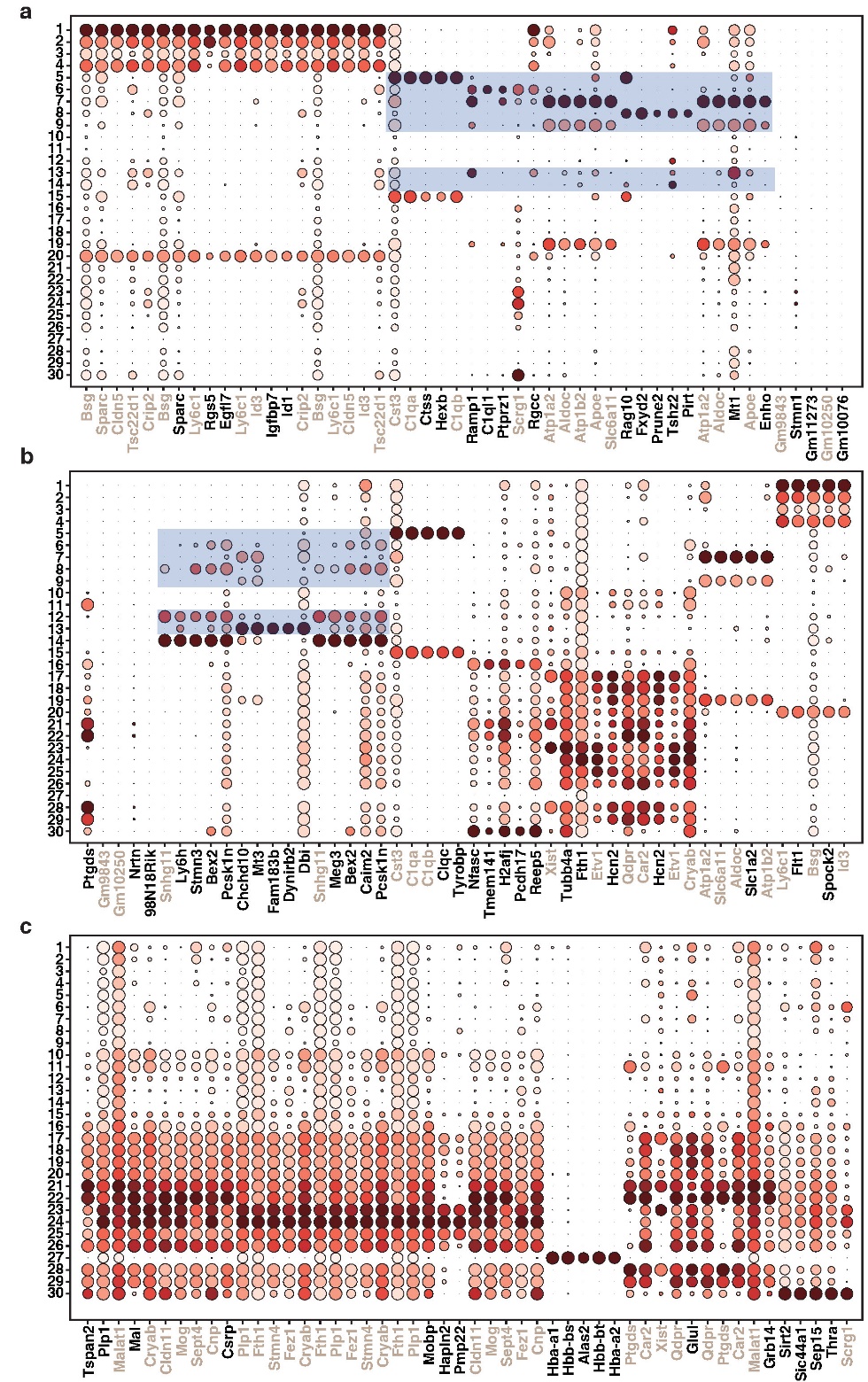
_

Supplementary Figure 5. Clusters of spinal cord cells detected by *k*NN+Louvain (*r* = 1.0). Top 5 marker genes of each cluster (at least 100 cells) are plotted as circles. Color darkness represents the mean expression of this gene (min-max normalized), and circle size represents the fraction of cells expressing this gene within the corresponding cluster. Genes belonging to a single cluster or multiple clusters are represented by black and light brown colors. Top 10 marker genes of clusters with light purple background (clusters 4 to 8, 12 to 13) are also illustrated in Fig. 3g.


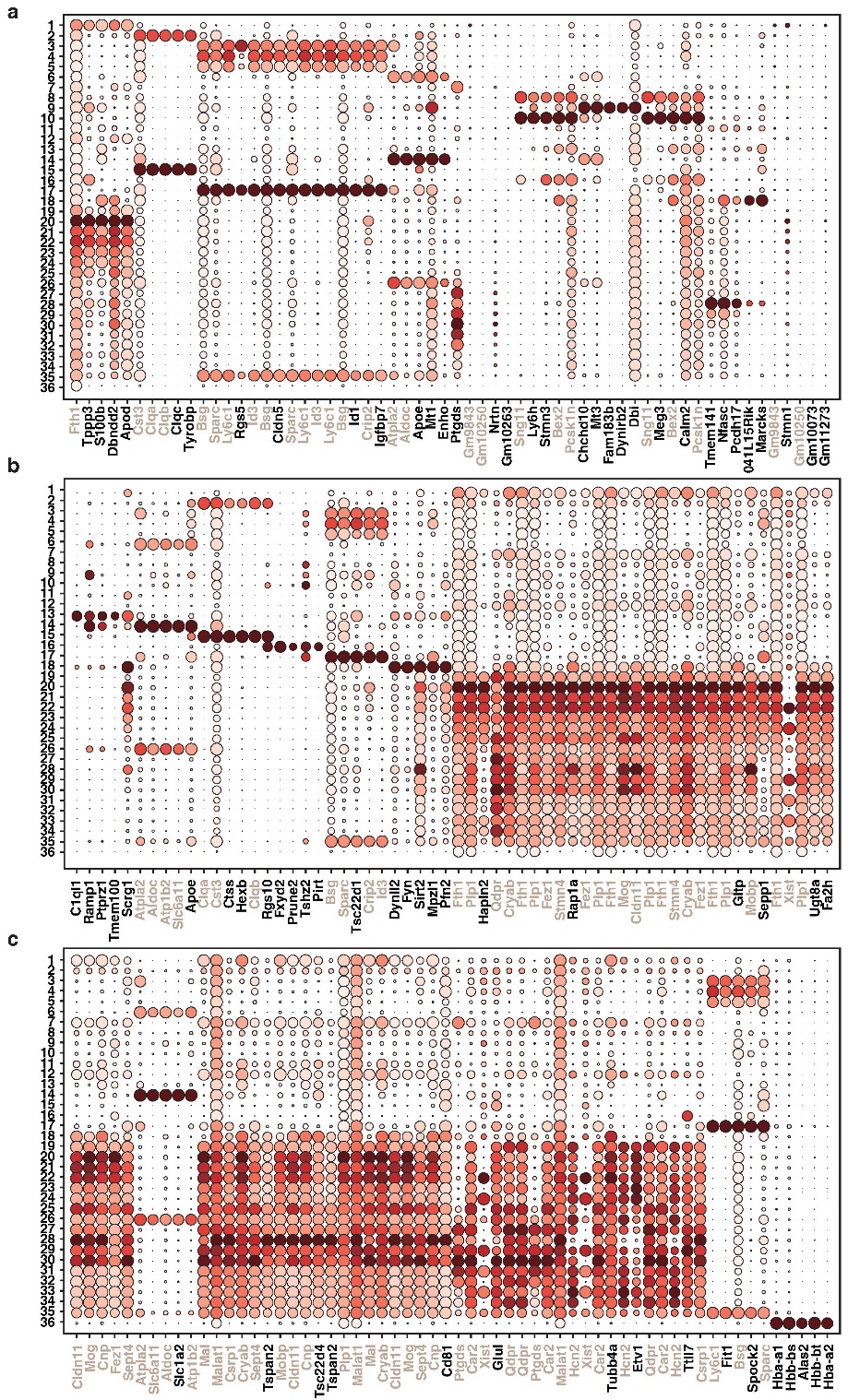


Supplementary Figure 6. Clusters of spinal cord cells detected by *k*NN+Louvain (*r* = 1.0). Top 5 marker genes of each cluster (at least 100 cells) are plotted as circles. Color darkness represents the mean expression of this gene (min-max normalized), and circle size represents the fraction of cells expressing this gene within the corresponding cluster. Genes belonging to a single cluster or multiple clusters are represented by black and light brown colors.


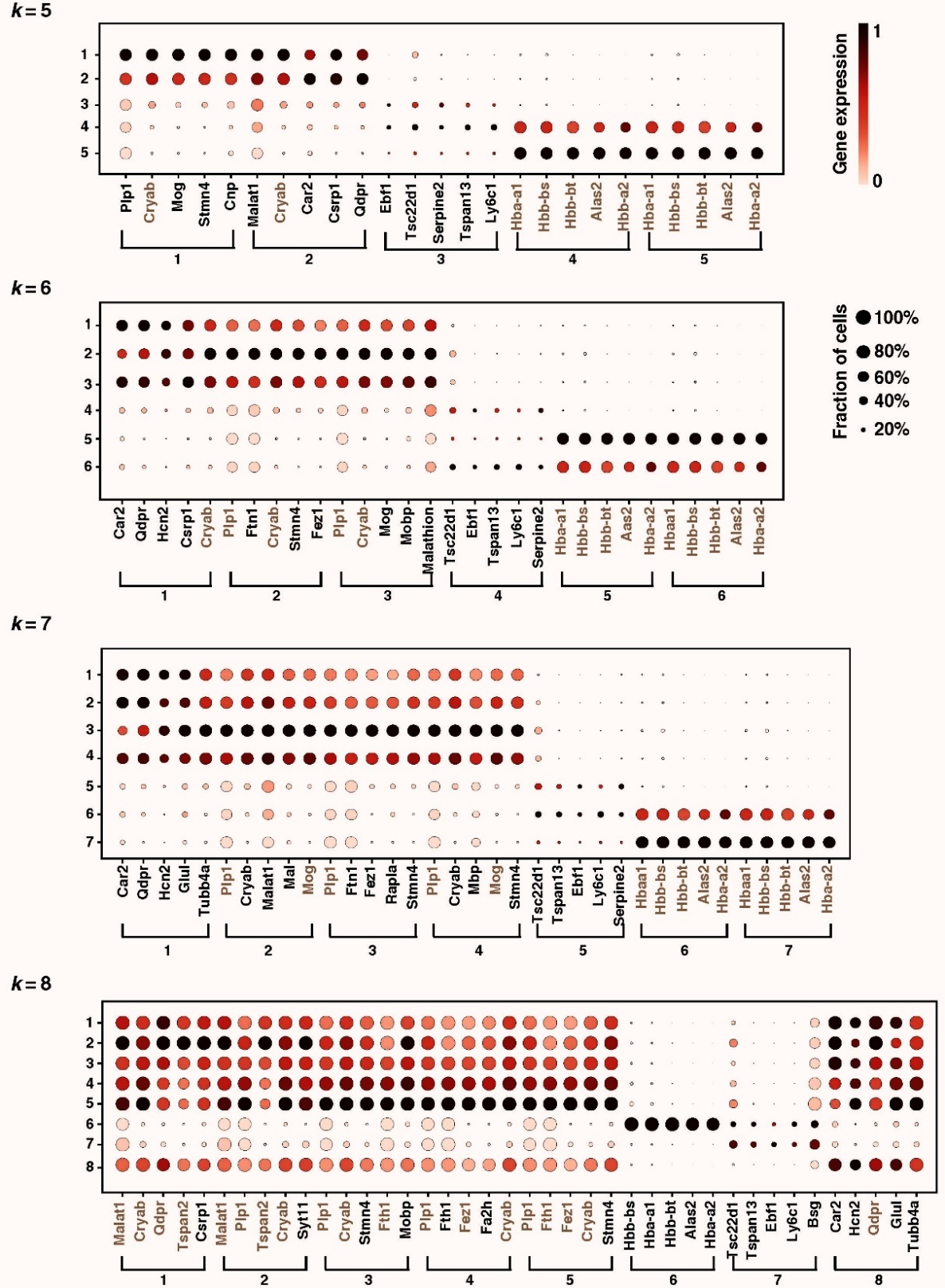


Supplementary Figure 7. Clusters of spinal cord cells detected by *k*-means. Top 5 marker genes of each cluster (at least 100 cells) are plotted as circles. Color darkness represents the mean expression of this gene (min-max normalized), and circle size represents the fraction of cells expressing this gene within the corresponding cluster. Genes belongs to a single cluster or multiple clusters are represented by black and light brown colors.

Supplementary Figure 8. Running time and usability of algorithms for clustering different numbers of images using centralized computing. WFC (Total) and WFC (Ave.) represent the total and average per-scale running times of WFC respectively. Algorithms fail to execute for given dataset sizes are not plotted.


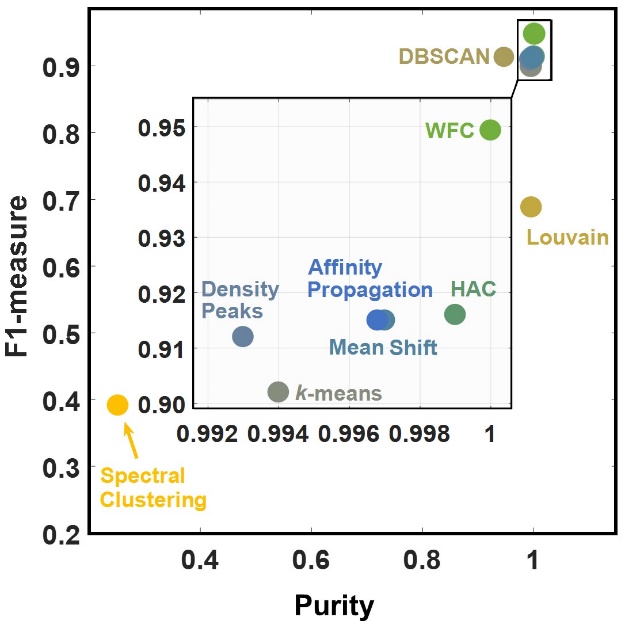


Supplementary Figure 9. Evaluation scores of clustering the first 5,000 HDFS logs. Each result was computed as an average by running each experiment 10 times.


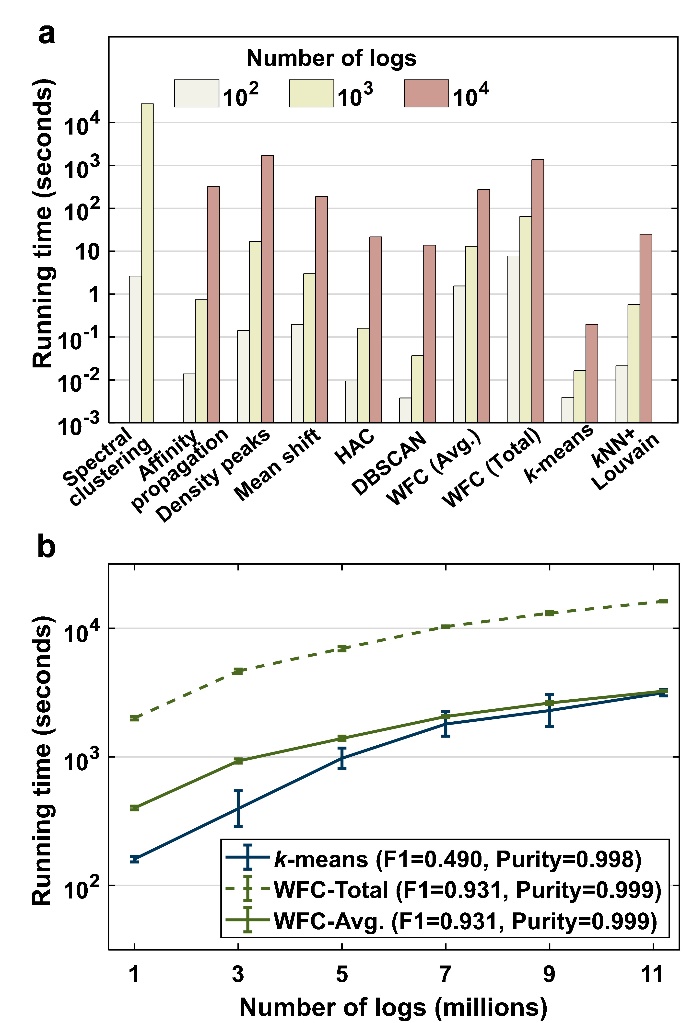


Supplementary Figure 10. Additional results of clustering HDFS logs. All results are the average of 10 runs of each algorithm. (a) Running time and usability of all algorithms for clustering different numbers of logs using centralized computing. WFC (Total) and WFC (Ave.) represent the total and average per-scale running times of WFC respectively. Algorithms fail to execute for given dataset sizes are not plotted. (b) Running times of WFC and k-means using distributed computing. Error bars indicate the standard error of the mean.


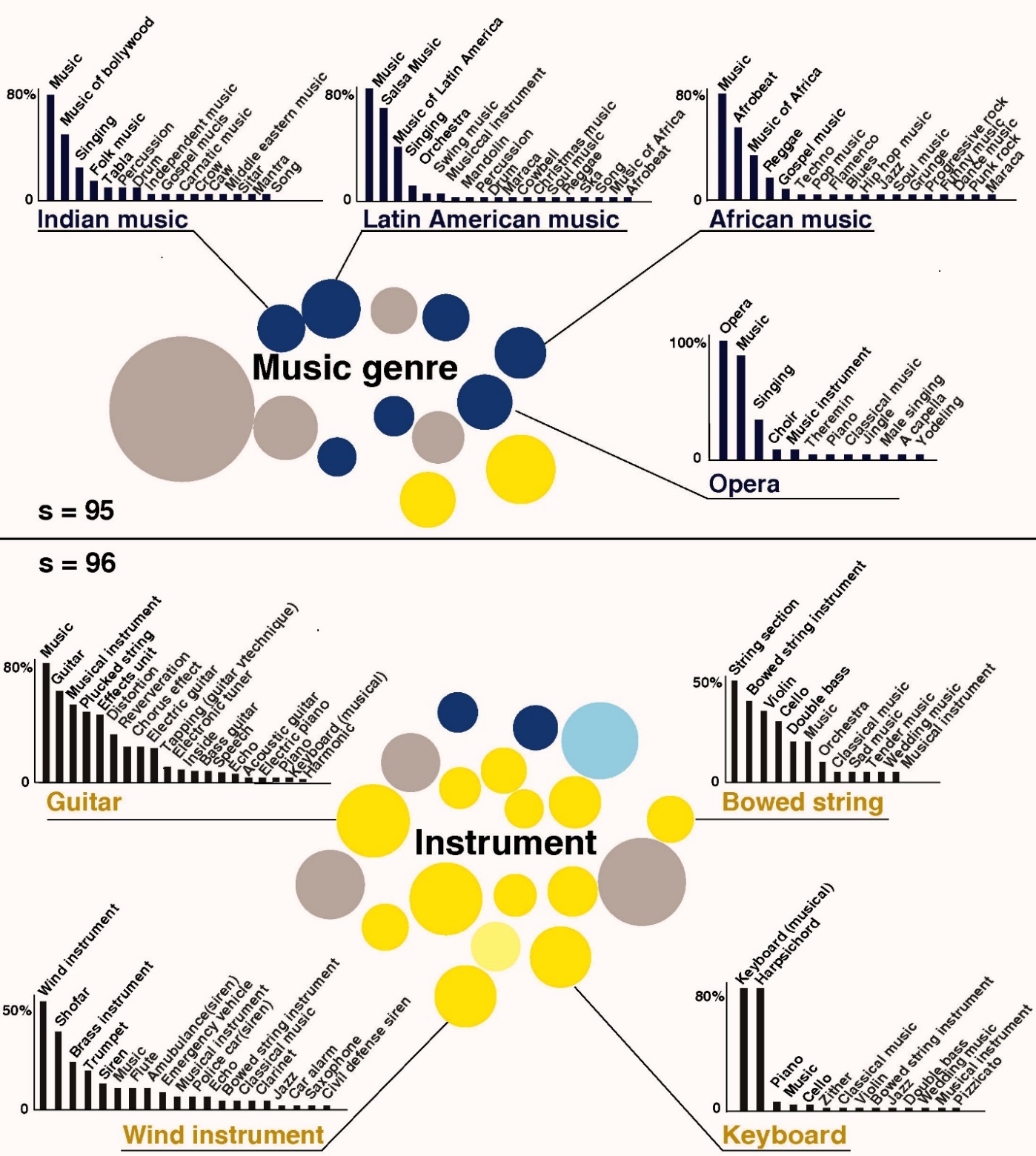


**Supplementary Figure 11.** **Clusters of music audios detected by WFC at scales 95 and 96**. For a given cluster and a label, denote the percentage of audios with the label with this cluster as the frequency of the label in this cluster. Circles represent detected clusters that contain at least 10 audios and their top-five frequent labels include “Music” or those related to instruments (e.g., “Guitar”). Blue, yellow and gray colors represent music genre styles, instrument types, and other audio clusters (e.g., plop) respectively. If a blue or yellow cluster has more than 50% audios labeled with “Music” or instrument-related labels, it is marked as dark; otherwise, light color. Bar graphs present the details of some clusters. Detailed results are listed in Supplementary Tables 9 and 10.


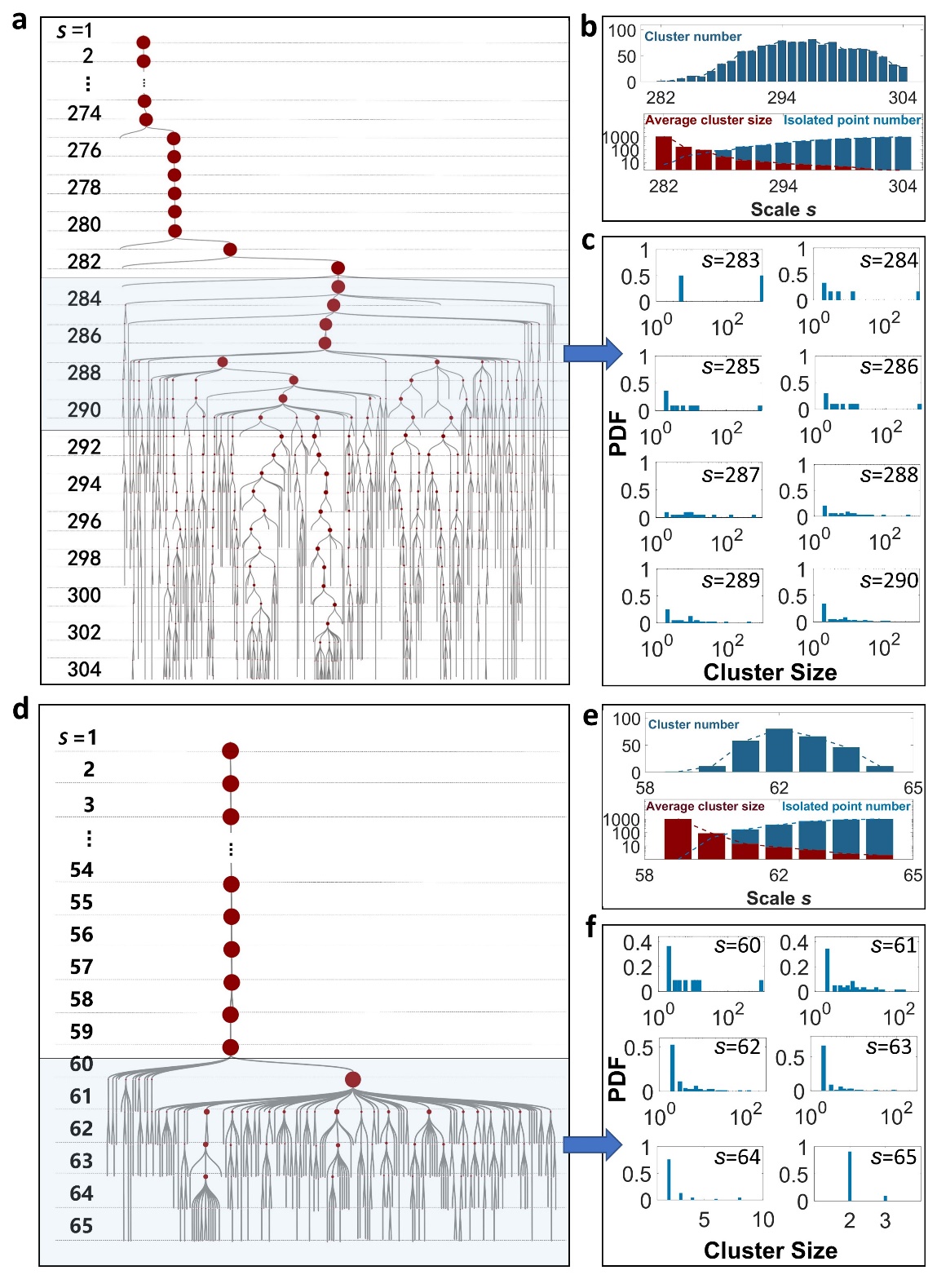


**Supplementary Figure 12. Visualization of clustering hierarchy and statistical results for the first 1,000 images in the CASIA-webface dataset, with λ=0.02 (a, b, and c) and λ=0.1 (d, e, and f).** (a and d) Visualization of clustering hierarchy. The radius of each brown circle (cluster) is proportional to the square root of the corresponding cluster size. For a clear visualization, each isolated data point *x* and the link to its parent cluster (the cluster containing x in the last scale) are not plotted. **(b and e)** Statistical results of clusters and isolated data points at typical scales (marked as light blue background). **(c and f)** Probability density function (PDF) of cluster sizes at each typical scale (marked as a light blue background).

**
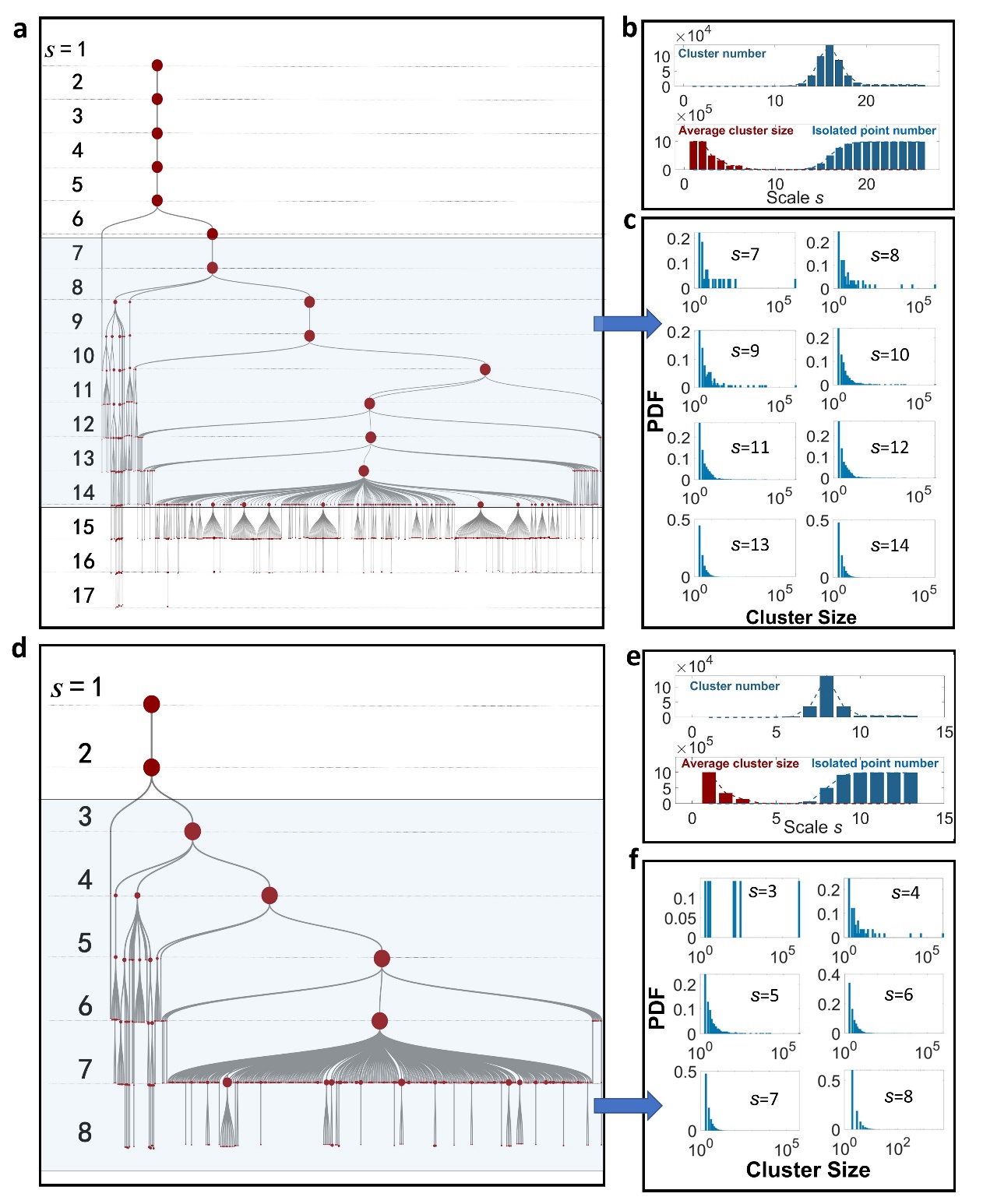
**

**Supplementary Figure 13. Visualization of clustering hierarchy and statistical results for the first a million locations in the NYC taxi dataset, with λ=1 (a, b, and c) and λ=3 (d, e, and f). (a and d)** Visualization of clustering hierarchy. The radius of each brown circle (cluster) is proportional to the cube root of the corresponding cluster size. For a clear visualization, all clusters with sizes smaller than 200 and isolated data points are not plotted. **(b and e)** Statistical results of clusters and isolated data points at a range of scales with significant clustering structures. **(d and f)** Probability density function (PDF) of cluster sizes at each typical scale (marked as a light blue background).

**Supplementary Table 1 Configurations of centralized and distributed computing environments**

| **Centralized Computing (Stand-alone)** | | |
| --- | --- | --- |
| **Hardware** | Memory | 126GB |
|  | CPU | Intel^®^ Xeon^®^ CPU E5-2640 v4 @ 2.40GHz 40 cores |
|  | Solid State Drive | 512GB |
|  | Hard Disk Drive | 4TB |
| Software | MATE Desktop Environment 1.12.1，Python2.7.4, DataSketches1.2.5, Networkx1.9 | |
| **Distributed Computing (Four servers)** | | |
|  | Memory | 512GB |
|  | CPU | Intel^®^ Xeon^®^ CPU E5-2640 v4 @ 2.40GHz 48 cores |
|  | Solid State Drive | 512GB |
|  | Hard Disk Drive | 7.6TB |
| **Software** | Ubuntu Server 16.04.3 LTS , Spark 2.1.2, Hadoop2.7.4, GraphLab Create2.1 | |

**Supplementary Table 2 Publicly available codes of all clustering algorithms discussed in the experiment.**

| **Algorithms** | **Python Codes** | **Spark Codes** |
| --- | --- | --- |
| WFC | <https://github.com/IoTDATALab/WFC> | <https://github.com/IoTDATALab/WFC/tree/master/spark> |
| *k*-means | <https://scikit-learn.org/stable/> | <https://spark.apache.org/mllib/> |
| DBSCAN |  | <https://github.com/irvingc/dbscan-on-spark> |
| Spectral |  | <https://github.com/windhaunting/bipartite-graph-coclustering> |
| Mean-shift |  | -- |
| Affinity Propagation |  | https://github.com/viirya/SparkAffinityPropagation |
| HAC |  | https://github.com/yu-iskw/hierarchical-clustering-with-spark |
| Density Peaks | https://github.com/CaoAo/DensityCluster |  |
| *k*NN+Louvain | <https://scanpy.readthedocs.io/en/stable/> | ***k***NN:  <https://github.com/saurfang/spark-knn> |
|  |  | Louvain:  <https://github.com/Sotera/spark-distributed-louvain-modularity> |

**Supplementary Table 3 Parameters settings in the experiment of clustering NYC taxi locations.**

| **Algorithm** | **Centralized Computing** | **Distributed computing** |
| --- | --- | --- |
| WFC | λ=1 | λ=1 |
| *k*-means | *k*=2, 3, 10, 100 (Fig. 2g) | *k* starts from 100 and increases by 100 every 50 million data points |
| Spectral clustering |  | N/A |
| HAC |  | N/A |
| Mean-shift | bandwidth = 0.01 | N/A |
| Density Peaks | sample_ratio = 0.01 | N/A |
| DBSCAN | (eps, mpt) =(0.05,5),(0.01,50) (Fig. 2g) | N/A |
| Affinity Propagation | damping = 0.9, preference = -0.001 | N/A |
| *k*NN+Louvain | *r* = 1.0; *k*=30, 60, 100, 200, 500 (Fig. 2g) | *k*=50, TreeSize=100, LeafSize=5 |

**Supplementary Table 4 Parameters settings in the experiment of clustering single cells of mouse nervous system.**

| **Algorithm** | **Centralized Computing** | **Distributed computing** |
| --- | --- | --- |
| WFC (all experiments) | N/A | λ=0.1 |
| *k*-means (all cells) | N/A | *k* =10,11, …, 38 |
| *k*NN+Louvain (all cells) | N/A | *r* =1.0, 1.5, 2.0; *k*=15 |
| *k*-means (spinal cord) | N/A | *k***=**5,6,7,8 |
| *k*NN+Louvain (spinal cord) | N/A | *r* =1.0, 1.5; *k*=15 |

**Supplementary Table 5. Parameters settings in the experiment of clustering face images.**

| **Algorithm** | **Centralized Computing** | **Distributed computing** |
| --- | --- | --- |
| WFC | λ=0.02 | λ=0.02 |
| *k*-means | *k* =5, 20, 200, 2000 (Supplementary Fig. 9) | *k* starts from 1,000 and increases by 17 for every 30 thousand data points |
| Spectral clustering |  | N/A |
| HAC |  | N/A |
| Mean-shift | bandwidth = 29 | N/A |
| Density Peaks | sample_ratio =0.01,0.0005 (Supplementary Fig. 9) | N/A |
| DBSCAN | (eps, mpt) = (21,3) | N/A |
| Affinity Propagation | damping = 0.9, preference =-8000 | N/A |
| *k*NN+Louvain | *r* =1.0; *k*=10 | *k*=10; TreeSize=100; LeafSize=5 |

**Supplementary Table 6 Parameters settings in the experiment of clustering HDFS logs.**

| **Algorithm** | **Centralized Computing** | **Distributed computing** |
| --- | --- | --- |
| Function:  gensim.models.word2vec.  Word2Vec | iter =50, size=30, min_count=2, window=5, all other parameters are set as their default values. | |
| WFC | λ=0**.**05 | λ=0.05 |
| *k*-means | *k* =2, 3, 5, 15, 50 (Supplementary Fig. 10a) | *k* starts from 5 and increases by 5 for every 1,000 data points |
| Spectral clustering |  | N/A |
| HAC |  | N/A |
| Mean-shift | bandwidth=150, 100, 90 (Supplementary Fig. 10a) | N/A |
| Density Peaks | sample_ratio = 0.01 | N/A |
| DBSCAN | (eps, mpt) = (50,15), (50,30), (60,50) (Supplementary Fig. 10a) | N/A |
| Affinity Propagation | damping = 0.9, preference =-20000 | N/A |
| *k*NN+Louvain | *r* =1.0; *k*=5, 30, 200, 2000 (Supplementary Fig. 10a) | N/A |

**Supplementary Table 7 Samples of system log texts in SOSP 2009 datasets**

| **Time tag** | **Type** | **Source namespace** | **Operation details** |
| --- | --- | --- | --- |
| 081110 010146 34 | INFO | dfs.FSNamesystem | BLOCK* NameSystem.addStoredBlock: addStoredBlock request received for blk_74623038764341097 on 10.251.201.204:50010 size 67108864 But it does not belong to any file. |
| 081110 010209 34 | INFO | dfs.FSNamesystem | BLOCK* NameSystem.addStoredBlock: addStoredBlock request received for blk_74623038764341097 on 10.251.123.33:50010 size 67108864 But it does not belong to any file. |
| … | … | … | … |

**Supplementary Table 8 Labels of logs in the SOSP 2009 dataset.**

| **Label** | **Type** | **Source name: Operation details** | **Number of logs** |
| --- | --- | --- | --- |
| 1 | INFO | dfs.DataNode$DataXceiver: Receiving | 1,723,232 |
| 2 | INFO | dfs.FSNamesystem: BLOCK | 3,721,054 |
| 3 | INFO | dfs.DataNode$PacketResponder: PacketResponder | 1,706,836 |
| 4 | INFO | dfs.DataNode$PacketResponder: Received | 1,706,514 |
| 5 | INFO | dfs.DataNode$DataXceiver: Received | 7,097 |
| 6 | INFO | dfs.DataNode$DataTransfer: IP | 6,937 |
| 7 | INFO | dfs.DataNode: IP | 7,002 |
| 8 | INFO | dfs.DataNode$DataXceiver: IP | 428,726 |
| 9 | INFO | dfs.DataBlockScanner: | 120,036 |
| 10 | INFO | dfs.DataNode$DataXceiver: writeBlock | 3,416 |
| 11 | INFO | dfs.FSDataset: Deleting block | 1,402,047 |
| 12 | INFO | dfs.DataNode$BlockReceiver: Receiving | 1,464 |
| 13 | INFO | dfs.DataNode$BlockReceiver: Exception | 155 |
| 14 | WARN | dfs.FSNamesystem | 975 |
| 15 | INFO | dfs.DataNode$BlockReceiver: Changing | 65 |
| 16 | INFO | dfs.DataNode$BlockReceiver: IP | 34 |
| 17 | ERROR | dfs.DataNode | 258 |
| 18 | WARN | dfs.DataNode$DataTransfer | 9 |
| 19 | WARN | dfs.FSDataset | 5,545 |
| 20 | WARN | dfs.DataNode$DataXceiver | 356,207 |
| 21 | INFO | dfs.FSDataset: Reopen Block | 5 |
| 22 | WARN | dfs.PendingReplicationBlocks | 47 |
| 23 | WARN | dfs.DataNode | 21 |
| 24 | WARN | dfs.DataBlockScanner | 10 |
| 25 | ERROR | du: cannot access | 13 |

**Supplementary Table 9 Clusters of different genre styles detected by WFC at** $\boldsymbol{s}\mathbf{=95}$**.**

| **Clusters (**$\boldsymbol{s}\mathbf{=95}$**)** | **Size** | **Top-5 frequent labels** |
| --- | --- | --- |
| Opera | 30 | Opera (24), Music (21), Singing (8), Choir (2), Theremin (2) |
| Indian music | 19 | Music (16), Music of Bollywood (10), Singing (5), Folk music (3), Tabla (2) |
| African music | 24 | Music (19), Afrobeat (13), Music of Africa (8), Reggae (4), Gospel music (2) |
| Latin America music | 38 | Music (29), Salsa music (24), Music of Latin America (14), Singing (4), Orchestra (2) |
| Happy music | 11 | Music (11), Happy music (3), Jingle (2), Music for children (2), Speech(1) |
| Electronica music | 18 | Music (15), Drum machine (4), Rhythm and blues (2), Harmonic (2), Electronica (2) |
| Flamenco music | 12 | Flamenco (12), Music (11), Singing (4), Speech (2), Yodeling (2) |
| Wind instrument | 31 | Wind instrument (31), Bagpipes (31), Drum (1), Percussion (1) |
| Percussion | 72 | Percussion (51), Drum (41), Bass drum (36), Snare drum (35), Rimshot (34) |
| Plop | 18 | Only three qualified labels: Speech (18), Plop (18), Music (1) |
| Telephone | 25 | Speech (6), Music (6), Telephone (4), Synthetic singing (3), Radio (3) |
| Background | 54 | Music (8), Silence (8), Speech (4), Busy signal (3), Plop (3) |
| Mixed audios | 8061 | Speech (1087), Music (1440), Inside (376), Animal (340), Vehicle (252) |

Each listed cluster contains at least 10 audios and their top-five frequent labels include “Music” or those related to instruments (e.g., “Guitar”).

**Supplementary Table 10 Clusters detected by WFC at** $\boldsymbol{s}\mathbf{=96}$**.**

| **Clusters (**$\boldsymbol{s}\mathbf{=96}$**)** | **Size** | **Top 5 frequent labels** |
| --- | --- | --- |
| Guitar | 92 | Music (85), Guitar (65), Musical instrument (55), Plucked string instrument (50), Effects unit (48) |
| Bowed string | 18 | String section (10), Bowed string instrument (8), Violin (7), Cello (6), Double bass (4) |
| Keyboard | 44 | Keyboard (40), Harpsichord (40), Piano (3), Music (2), Cello (2) |
| Plucked string | 25 | Pizzicato (22), Harp (21), Zither (3), Singing bowl (1), Plucked string instrument (1) |
| Didgeridoo | 13 | Only three qualified labels: Didgeridoo (13), Music (13), Musical instrument (1) |
| Wind instruments | 46 | Wind instrument (25), Shofar (18), Brass instrument (11), Trumpet (9,) Siren (6) |
| Harmonica | 22 | Only two qualified labels: Harmonica (22) and Wind instrument (22) |
| Bagpipes | 14 | Only four qualified labels: Wind instrument (14), Bagpipes (14), Drum (1), Percussion (1) |
| Rapping | 17 | Music (13), Rapping (9), Hip hop music (4), Rhythm and blues (2), Reggae (2) |
| Sitar | 18 | Sitar (10), Music (10), Tabla (8), Percussion (8), Drum (7) |
| Bell | 89 | Change ringing (45), Church bell (31), Bell (15), Music (6), Tubular bells (6) |
| Mallet percussion | 21 | Music (9), Glockenspiel (8), Marimba (8), Mallet percussion (7), Ding-dong (3) |
| Rock music | 12 | Music (11), Punk rock (5), Rock music (4), Progressive rock (4), Musical instrument (2) |
| Opera | 16 | Opera (15), Music (8), Singing (4), Jingle (1) |
| Ambient music | 116 | Only four qualified labels: Music (50), Ambient music (19), Gong (15), Organ (14) |
| Buzz | 15 | Electric shaver (10), Speech (7), Inside (7), Music (3), Buzz (2) |
| Background 1 | 38 | Music (6), Silence (5), Plop (3), Busy signal (3), Outside (2) |
| Background 2 | 230 | Speech (45), Music (26), Television (9), Knock (9), Slap (8) |
| Background 3 | 17 | Speech (15), Applause (3), Chuckle (3), Snicker (3), Conversation (2) |

Each listed cluster contains at least 10 audios and their top-five frequent labels include “Music” or those related to instruments (e.g., “Guitar”).
